# Simulating Porous Magnetite Layer Deposited on Alloy 690TT Steam Generator Tubes

**DOI:** 10.3390/ma11010062

**Published:** 2018-01-02

**Authors:** Soon-Hyeok Jeon, Yeong-Ho Son, Won-Ik Choi, Geun Dong Song, Do Haeng Hur

**Affiliations:** Nuclear Materials Research Division, Korea Atomic Energy Research Institute, Daejeon 305-353, Korea; junsoon@kaeri.re.kr (S.-H.J.); syhm0907@kaeri.re.kr (Y.-H.S.); wonik134@kaeri.re.kr (W.-I.C.); sgd84@kaeri.re.kr (G.D.S.)

**Keywords:** electrodeposition, magnetite, steam generator, flake, sludge

## Abstract

In nuclear power plants, the main corrosion product that is deposited on the outside of steam generator tubes is porous magnetite. The objective of this study was to simulate porous magnetite that is deposited on thermally treated (TT) Alloy 690 steam generator tubes. A magnetite layer was electrodeposited on an Alloy 690TT substrate in an Fe(III)-triethanolamine solution. After electrodeposition, the dense magnetite layer was immersed to simulate porous magnetite deposits in alkaline solution for 50 days at room temperature. The dense morphology of the magnetite layer was changed to a porous structure by reductive dissolution reaction. The simulated porous magnetite layer was compared with flakes of steam generator tubes, which were collected from the secondary water system of a real nuclear power plant during sludge lancing. Possible nuclear research applications using simulated porous magnetite specimens are also proposed.

## 1. Introduction

Magnetite formed in the feed water, condensate, and the drain systems is deposited on the surfaces of steam generator (SG) tubes, the top of tube sheets, and the tube support structure in secondary systems in the pressurized water reactors (PWRs) of nuclear power plants [1,2].

Magnetite deposited on the outside of SG tubes can limit the heat exchange capability of an SG either by decreasing the heat transfer efficiency or by interfering with the SG hydrodynamics. In addition, aggressive chemical impurities, such as chloride ions, sulfate ions, and lead, can concentrate in porous magnetite deposits [3,4,5] and thus induce corrosion degradation of SG tubes, such as intergranular attack, pitting, denting, thinning, and stress corrosion cracking [5,6,7,8,9,10]. For these reasons, there has been intense interest in magnetite accumulated on SG structural materials because its removal improves heat transfer efficiency and mitigates the corrosion problems of SG tubes. To investigate the corrosion behavior of SG materials that are deposited the magnetite and dissolution of the magnetite, a proper method to deposit magnetite on SG material substrates should be established.

In a previous study, Sapieszko and Matijevic [11] reported the cathodic reduction of Fe^3+^ complexed with triethanolamine (TEA) to produce pure magnetite by the hydrothermal method. In recent years, Switzer and others have modified the Fe(III)-TEA solution agent to more easily deposit magnetite on stainless steel [12], Au [13], and Ni substrates [14]. Furthermore, Goujon et al. [15,16] established the optimal electrodeposition conditions to produce thick and dense magnetite films on nickel-based alloys in an Fe(III)-TEA solution. Duan et al. [17] presented a systematic study regarding factors affecting magnetite electrodeposition, such as the concentration of Fe(III) ions as well as the deposition temperature and time in the Fe(III)-TEA solution. They showed that the Fe(III) ion concentration had an impact on the deposition rate, while the deposition time and temperature strongly affected the morphology of magnetite films. However, so far, there have been no investigations on the production of porous magnetite layers by electrodeposition in an Fe(III)-TEA solution.

To elucidate the corrosion behaviors of real corrosion products that are deposited on SG tubes and the dissolution of corrosion products in secondary water systems, it is necessary to establish a proper method to produce a porous magnetite layer as well as an adherent magnetite layer because real magnetite deposited on SG tubes in an operating SG has a porous structure [18].

This paper introduces a modified method to simulate porous magnetite that is deposited on the surface of SG tubing in the secondary system of a nuclear power plant. The magnetite layers were electrodeposited on Alloy 690 substrates at −1.05 V_SCE_ in an Fe(III)-TEA solution at 80 °C. After electrodeposition, the magnetite layers were subjected to an immersion treatment to produce porous magnetite by reductive dissolution reaction in an alkaline solution. This modified method is a new approach to simulate the porous magnetite deposited on the SG tubes in nuclear power plants by electrodeposition. In addition, this method is a very simple and easy way to produce porous magnetite because the deposition and immersion solution temperatures are lower than 80 °C.

In addition, the flakes collected from an SG of a real nuclear power plant during sludge lancing were characterized for comparison to the electrodeposited magnetite layer by high-resolution X-ray diffraction (HR-XRD) and a scanning electron microscope (SEM) equipped with an energy-dispersive X-ray spectrometer (EDS), and an electron back-scatter diffraction (EBSD) pattern analyzer. The porosity, pore size, and distribution were measured from the cross-sectional images of the flakes by image analysis. We also propose nuclear research applications using simulate porous magnetite specimens electrodeposited on Alloy 690 SG tubes in the secondary water system of nuclear power plants.

## 2. Materials and Methods

Alloy 690 was melted in a high-frequency vacuum-induction furnace and hot-rolled at temperatures in a range of 1250–1150 °C. The plates were cold-rolled with a total area reduction of about 70%. The specimens were heat processed in two steps, namely, mill annealing (MA) and thermal treatment, after being cold-rolled. The cold-worked samples were mill annealed at 1100 °C for 5 min, followed by water quenching, and then thermally treated at 715 °C for 10 h in a vacuum furnace under a pressure of about 5 × 10^−6^ Torr. Alloy 690 plates were machined into specimens 10 × 5 × 1 mm^3^. The Alloy 690 substrates were wet-ground with silicon carbide papers down to # 1000 grits, and then ultrasonically cleaned in ethanol for 5 min. The chemical composition of Alloy 690TT was analyzed by the inductively coupled plasma-mass spectroscopy (ICP-MS, Agilent, Santa Clara, CA, USA). The chemical composition of Alloy 690TT is given in Table 1.

For comparison with the magnetite layers electrodeposited on an Alloy 690TT substrate, flakes were collected from the SG tubes of a real nuclear power plant during sludge lancing. The elemental content of the flake samples was examined by X-ray fluorescence (XRF) spectrometry using a Rigaku ZSX Primus II (Rigaku, The Woodlands, TX, USA). The element content of the flake samples is given in Table 2.

Magnetite layers were electrodeposited from an alkaline solution of Fe_2_(SO_4_)_3_ complexed with TEA. The concentrations in the electrodeposition bath were 0.09 M Fe_2_(SO_4_)_3_, 0.1 M TEA, and 2 M NaOH. The deposition solution was prepared by the slow addition of Fe_2_(SO_4_)_3_ dissolved in water to a stirred solution of NaOH and TEA, and the solution was degassed. The resulting deposition solution was gray-green colored with pH 12.5 at 25 °C. The resulting gray-green solution was heated to 353 K and stirred at 200 rpm.

Electrodeposition of the magnetite layers on the Alloy 690TT substrate was conducted using a PAR273 potentiostat (Ametek, Berwyn, PA, USA) with Power-Suite software (version 2.55, Schneider Electric, Rueil-Malmaison, France) and a three-electrode cell. A saturated calomel electrode (SCE) and a platinum wire were used as the reference and counter electrode, respectively. Magnetite layers were electrodeposited on the Alloy 690TT substrates in the prepared deposition solution at −1.05 V_SCE_ for 10 h. After electrodeposition, to simulate the porosity of magnetite that are deposited on SG tubes, the electrodeposited magnetite samples were immersed in an alkaline solution with a pH level of 9.5 at room temperature for 50 days.

The electrodeposited magnetite layers and flakes that were collected from an SG tube were analyzed to characterize their structure by HR-XRD analysis conducted with a Rigaku SmartLab with a two-dimensional (2D) detector (Hypix-3000, Rigaku, Tokyo, Japan). For the HR-XRD analysis, Cu-K_α1_ radiation was applied. The lattice constants of the samples were also calculated from the peak locations and Miller indices by JADE9 software (Material Data, Inc., Livermore, CA, USA). The full-width at half-maximum (FWHM) value is measured by the XRD diffraction patterns. According to the Sherrer’s formula [19], the crystallite size *D_hkl_* for the samples is given by Equation (1), as follows.

(1) $$D_{hkl}=\frac{K\times \lambda }{\beta \times \cos\theta }$$

where β is the FWHM value of XRD diffraction patterns, *K* is the unit cell geometry dependent constant (0.89), λ is the wavelength of the XRD (0.154056 nm), and θ is the half diffraction angle of 2θ. The average crystallite size is determined by taking the average of the sizes at the six peaks.

To observe the cross section of the samples, the samples were milled by a focused ion beam (FIB) directed vertically toward the magnetite layers and observed by SEM using a QUANTA 3D FEG FIB-SEM (FEI Company, Hillsboro, OR, USA). In addition, the chemical composition and structure of the samples were analyzed by SEM-EDS and EBSD. The porosity, pore size, and pore distribution were measured from the cross-sectional images of the flake and electrodeposited magnetite samples by image analysis (Image-Pro Plus 7.0, Media Cybernetics, Rockville, MD, USA).

## 3. Results

### 3.1. Electrodeposition of the Magnetite Layers

**Figure 1:**
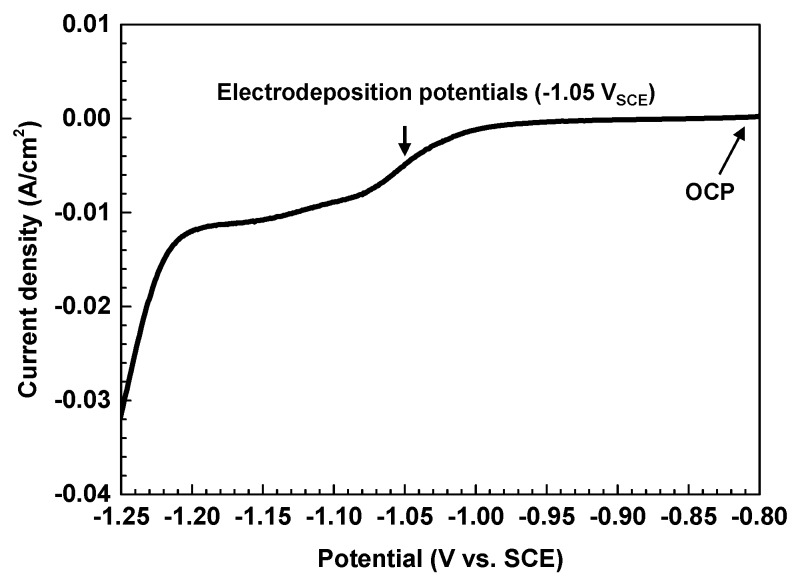
Linear sweep voltammogram of Alloy 690TT substrate in Fe(III)-TEA solution at 80 °C.

Figure 1 shows the linear sweep voltammogram obtained for the Alloy 690TT substrate in an Fe(III)-TEA bath at 80 °C. The potential was swept from the open circuit potential to −1.25 V_SCE_ at a scan rate of 1 mV/s. The electrochemical reduction of Fe(III)-TEA started at potentials more negative than −0.80 V_SCE_. The first reduction wave of this linear sweep was measured to be approximately between −0.95 and −1.20 V_SCE_. The second reduction wave was also observed in the potential range from −1.20 to −1.25 V_SCE_. The electrodeposition of magnetite layers in an Fe(III)-TEA solution can be simplified by two processes. In the first process, the Fe(III)-TEA solution is electrochemically reduced to an Fe(II)-TEA complex. The reaction that may occur is expressed as reaction (2). In the second process, the Fe(III)-TEA complex would then chemically react with Fe(II)-TEA to produce magnetite at the surface of the substrate in the subsequent reaction (3). The overall electrochemical reaction, leading to the formation of a magnetite deposit on the substrate, is reaction (4). The proposed mechanism is expressed as follows [12,13,14].

Fe(OH)_4_TEA_2_^−^ + e^−^ → Fe(TEA)_2_^2+^ + 4OH^−^ (2)

2Fe(OH)_4_TEA_2_^−^ + Fe(TEA)_2_^2+^ ↔ Fe_3_O_4_ + 6TEA + 4H_2_O (3)

3Fe(OH)_4_TEA_2_^−^ + e^−^ → Fe_3_O_4_ + 6TEA + 4H_2_O + 4OH^−^ (4)

In this study, the magnetite layers were electrodeposited on Alloy 690TT substrates at −1.05 V_SCE_ in an Fe(III)-TEA solution for 10 h at 80 °C.

**Figure 2:**
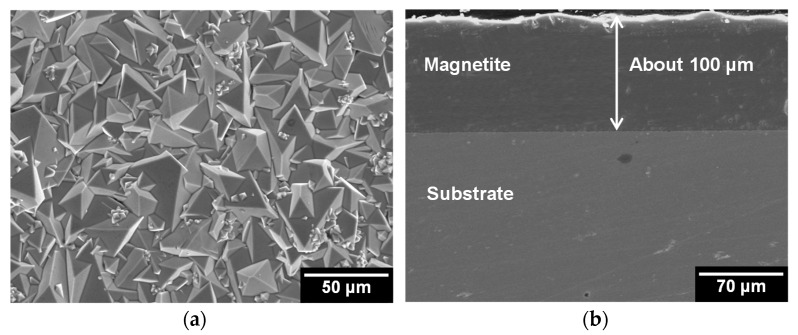
SEM images of the surface and cross section of electrodeposited magnetite layer on Alloy 690TT substrate: (**a**) surface and (**b**) cross section.

Figure 2 shows SEM images of the surface and cross section of the magnetite layers. The magnetite layers had highly faceted morphologies (Figure 2a). The diameters of the magnetite particles were in the range of about 3–25 μm. As shown in Figure 2b, the thickness of the magnetite layers was about 100 μm. The magnetite layers were very compact and adherent to the Alloy 690TT substrate. No defects, such as holes, gaps, or cracks could be observed at the interface between the magnetite layers, and the Alloy 690TT substrate, which confirms that the magnetite layers were tightly bonded to the Alloy 690TT substrate. In previous research [20], the surface concentration of Fe(III) is nearly the same as the bulk concentration because the ion diffusion is sufficient in this electrodeposition condition. Therefore, the structure is determined by the preferential growth of favorably oriented crystal faces. As a result, the electrodeposited film that is formed transforms to a dense and crack-free morphology with tightly packed, well-faceted crystallites. Hence, magnetite particles were observed as the triangle shape.

### 3.2. Comparison the Porous Magnetite Layers with Flakes Taken from an SG Tube

In the secondary water systems of nuclear power plants, the reductive dissolution of magnetite, and the formation of dissolved ferrous species occur under alkalized reducing conditions by the following reaction [21,22,23,24,25,26,27].

Cathodic reaction: Fe_3_O_4_ + 2H_2_O + 2H^+^ + 2e^−^ = 3Fe(OH)_2_ (5)

Anodic reaction: H_2_ = 2H^+^ + 2e^−^ (6)

Overall reaction: Fe_3_O_4_ + 2H_2_O + H_2_ = 3Fe(OH)_2_ (7)

To simulate the porosity of reductively dissolved magnetite deposits on SG tubing during SG operation, the electrodeposited magnetite layers were immersed in an alkaline solution with a pH level of 9.5 at room temperature for 50 days after electrodeposition.

In this work, the electrochemical reaction of magnetite could be divided into the two stages. In the first stage, dense magnetite is reductively dissolved by the oxidation reaction of hydrogen (hydrogen dissolved in water). Although hydrogen addition is not involved in the experimental procedure, there is always a small amount of hydrogen in water. Under these conditions, the reductive reaction rate is very slow because the amount of hydrogen that is involved in the reaction is very small. The second stage is when the surface of Alloy 690 substrate is exposed as the dense magnetite turn into the porous structure. In the second stage, the corrosion of metals, such as iron, chromium, and nickel does occur in the oxidation reaction to supply the electrons (M → M*^x^*^+^ + *x*e^−^), and hydrogen gas is generated in reduction reaction (2H_2_O + 2e^−^ → H_2_ (gas) + 2OH^−^). The dissolution reaction rate of magnetite will be increased due to the increase of hydrogen, as well as the increase of electrons due to the corrosion of the metals.

The porous magnetite layers were compared with the flakes that were obtained from an SG tubes of real nuclear power plant, which were collected from a secondary water system during sludge lancing. The element composition of the flake samples observed by XRF analysis is given in Table 2. As determined from the XRF data, the predominant elements in the flake samples were iron, nickel, and manganese. Small amounts of other elements (chromium, copper, zinc, titanium, and aluminium) were observed. The ratio of magnetite, trevorite, and chromite may have differed between the electrodeposited magnetite layer and the flakes due to differences between the dissolved element content of the test solution and those in the actual secondary water system from which the flakes were taken.

**Figure 3:**
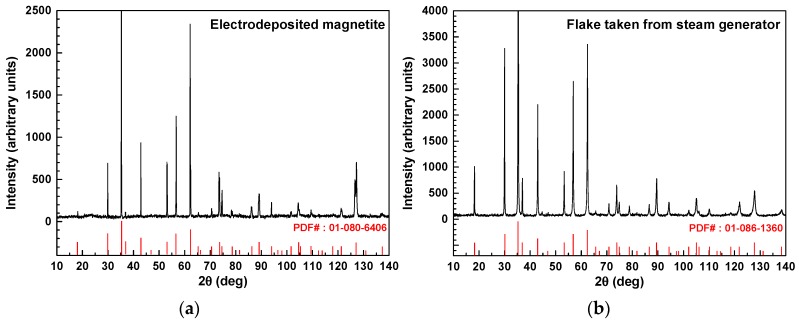
High-resolution X-ray diffraction (HR-XRD) patterns of the electrodeposited magnetite layer on Alloy 690TT substrate and flakes taken from SG tubes: (**a**) electrodeposited magnetite layer and (**b**) flakes.

To clarify the structure of the flakes and compare it with the electrodeposited magnetite layer, the samples were examined by HR-XRD and SEM equipped with an EBSD and EDS. XRD identification of phase in the samples was conducted prior to SEM-EDS and EBSD analyses. Figure 3 shows the HR-XRD patterns of dense magnetite layers on the Alloy 690TT substrate and flakes removed from an SG tube of an actual nuclear power plant. As shown in Figure 3a, the electrodeposited magnetite layer was highly crystalline, and the position and relative intensity of the diffraction peak of the sample matched well with the standard XRD data for magnetite (PDF No. 01-080-6406). The peaks of any other phases were hardly observed in HR-XRD patterns, indicating the high purity of the products. Figure 3b shows that the flake sample was also highly crystalline, and all of the peaks corresponded to magnetite (PDF No. 01-086-1360). In addition, the lattice constants of the electrodeposited magnetite and flake samples were calculated from peak locations and Miller indices by JADE9 software (version 9.0) (Table 3). Comparison with the PDF data (magnetite, trevorite, and chromite) showed that the lattice constants of the electrodeposited magnetite layer and flake samples were contained within the range of the lattice constants of the PDF data for magnetite (Table 3). The peak broadening of XRD pattern indicates that the crystallite size is small. The crystallite size could be calculated by the Sherrer’s formula. The FWMH and crystallite sizes of the electrodeposited magnetite layer and flake samples are given in Table 4. Average crystallite size of electrodeposited magnetite is larger than that of the flake samples by about 1.7 times.

Based on the diffraction patterns and lattice constants, the electrodeposited magnetite layer and flake samples were identified as magnetite. However, in the case of the flake samples, the small amount of other structures containing nickel, chromium (trevorite and chromite), or metallic copper particles can be formed due to the presence of trace amounts of nickel, chromium, and copper in the flakes. However, XRD analysis has a detection limit for small amounts of other phases due to the noise signal or slgiht shifting of diffraction patterns.

To solve these problems, the flake samples were ion-milled by a FIB in a vertical direction and analysed by SEM-EDS and EBSD to clarify the flake structure. First, point EDS analysis was conducted to determine the chemical composition at various particles of the electrodeposited magnetite and flake samples. The results of the quantitative point EDS analysis are given in Table 5. Points 1 and 2 corresponded to magnetite particles in the electrodeposited magnetite layer, respectively. In the case of the flake samples, point 3 represented a magnetite particle because it was composed of approximately 40.3 at % Fe and 59.7 at % O. Point 4 could represent a trevorite particle because it was composed of 37.1 at % Fe, 17.9 at % Ni, and 45.0 at % O. Point 5 represented metallic copper consisting of 5.7 at % Fe, 2.1 at % Ni, 86.4 at % Cu, and 5.8 at % O. The oxygen contents of the electrodeposited magnetite sample (points 1 and 2) and magnetite in the flake (point 3) were almost the same in the range of 57 at %–60 at %. However, there was a difference between the oxygen content of the electrodeposited magnetite and points 4 and 5 in the flake sample. The relatively low oxygen content exhibited at points 4 and 5 may be caused by the presence of trevorite and metallic copper. Based on the XRD and SEM-EDS results, the flakes were mostly composed of magnetite and contained small amounts of trevorite and metallic copper. Although the oxygen contents of the electrodeposited magnetite and trevorite and metallic copper were quite different, the electrodeposited magnetite sample effectively simulated the porous magnetite-based flakes formed on SG tubes in PWRs because the flakes were mostly composed of magnetite.

**Figure 4:**
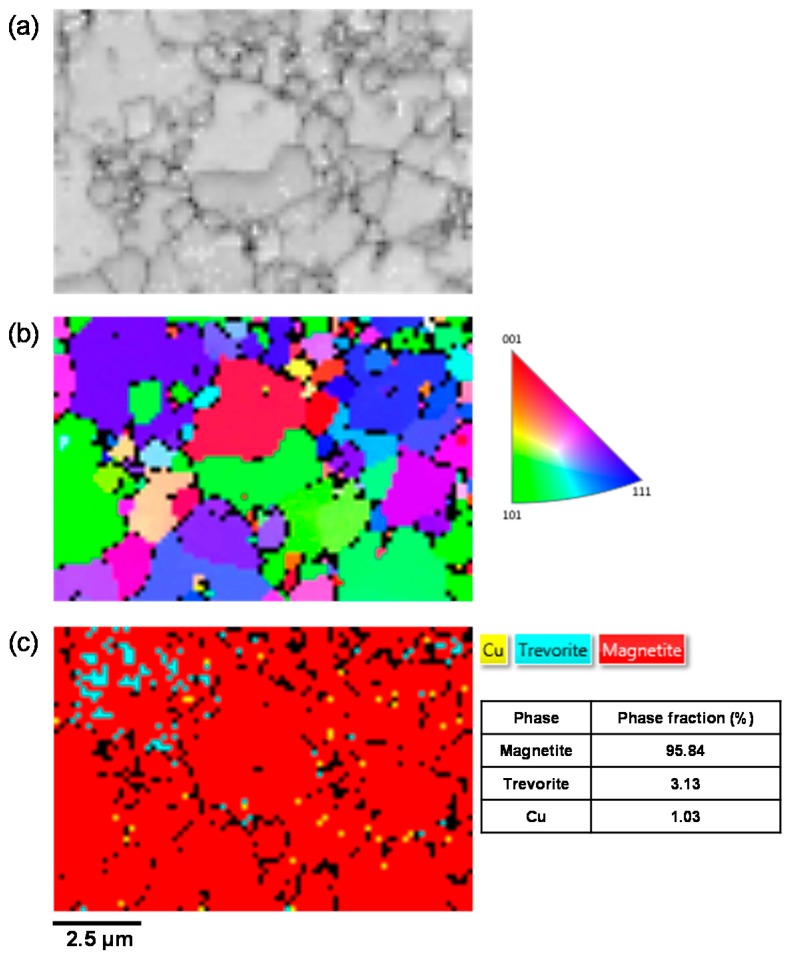
Electron back-scatter diffraction (EBSD) data of the flakes collected from steam generator (SG) tubes: (**a**) IQ index representation; (**b**) 001 IPF orientation map; and (**c**) phase map and relative phase fraction.

The EBSD analysis was performed to measure the phase fraction of the flake samples. Figure 4 shows the EBSD data of a flake collected from an SG tube. A first map based on the image quality pattern (IQ) data (Figure 4a) reveals a contrast that is related to local variations of surface quality, crystallinity, and orientation. In the 001 inverse pole figure (IPF) orientation map (Figure 4b), a random orientation is observed (no predominant color appears). The phase distribution map (Figure 4c) confirms that the flake sample contained three phases composed of 95.84% magnetite in surface proportion with 3.13% trevorite and 1.03% metallic copper. These results indicate that the flake samples were composed mainly of magnetite and small amounts of trevorite and metallic copper. The elemental concentrations, as determined by XRF, were consistent with the SEM-EDS and EBSD data. The electrodeposited magnetite that was produced in this work could not perfectly simulate the flakes, which included not only magnetite (about 96%) but also various compounds, such as trevorite (about 3%) and Cu (about 1%). However, because the flakes consisted of over 95 % magnetite and had a porous structure, the electrodeposited magnetite is sufficiently similar to simulate the flakes on the SG tubes of actual PWRs.

**Figure 5:**
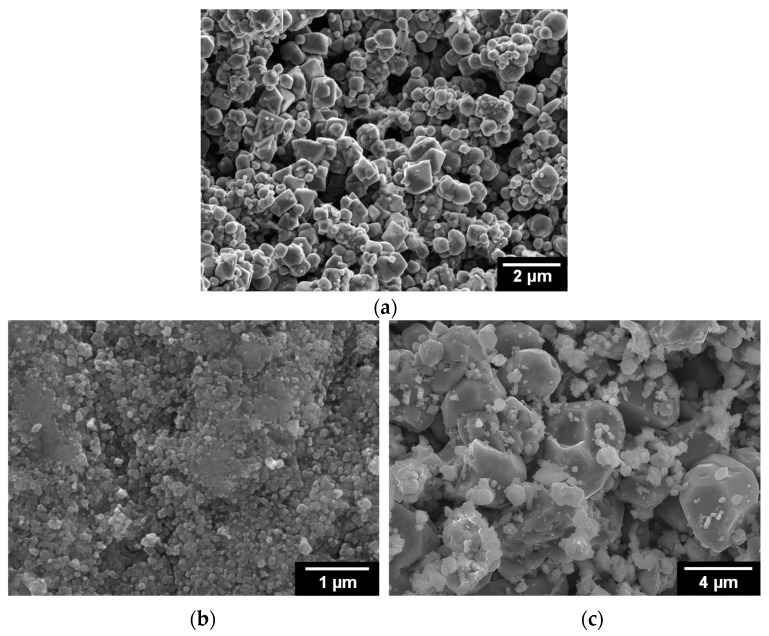
SEM images of the magnetite layer after immersion in an alkaline solution for 50 days at room temperature and flakes collected from SG tubes: (**a**) magnetite layer; (**b**) tube side of flake; and (**c**) water side of flake.

Figure 5 shows SEM images of the magnetite layers after immersion treatment and the flakes that were taken from the SG tube. The electrodeposited magnetite particles were reductively dissolved in an alkaline solution. In comparison to the magnetite particles before immersion treatment, relatively round magnetite particles of sizes in the range of 30–800 nm with numerous small pores were observed. Figure 5b,c shows the tube side and water side of the flakes, respectively. Although the round morphologies of both sides of the flakes were very similar, the size of the particles on the tube side of the flakes was significantly different from that of particles on the water side of the flakes. The tube side of the flakes was composed of extremely small particles (Figure 5b). Numerous small pores were observed between the small magnetite particles. As shown in Figure 5c, the water side of the flakes was composed of relatively coarse particles, which resulted in the formation of large pores.

**Figure 6:**
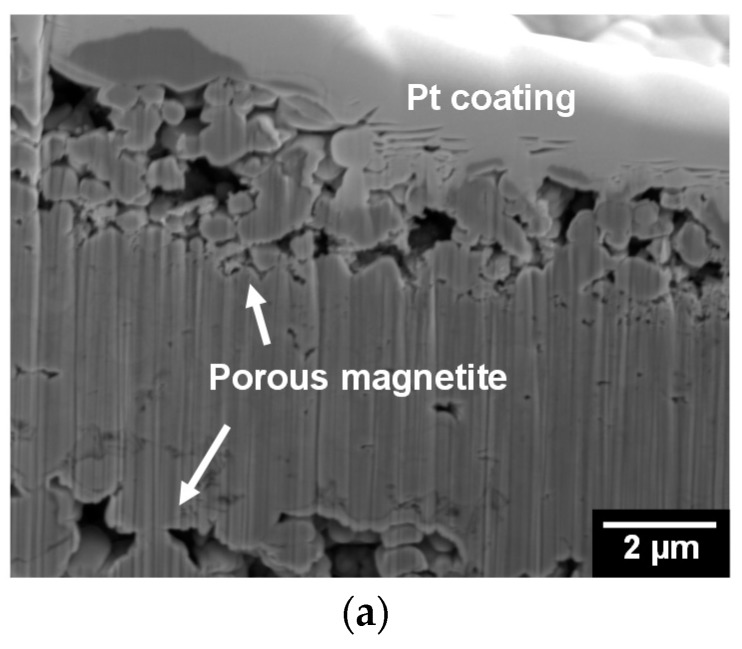

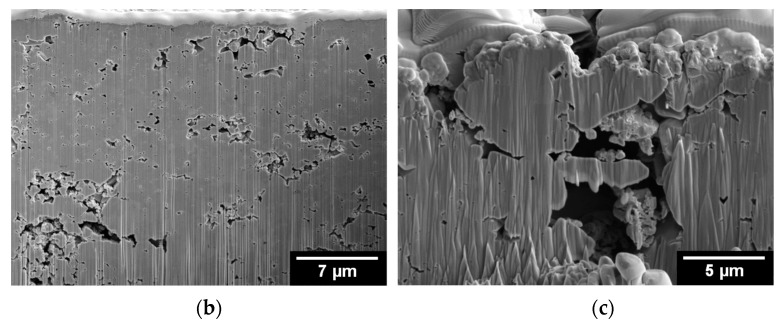
Focused ion beam (FIB)-SEM images of the magnetite layer after immersion in an alkaline solution for 50 days at 25 °C and flakes collected from SG tubes: (**a**) magnetite layer; (**b**) tube side of flake; and (**c**) water side of flake.

Figure 6 shows FIB-SEM images of the magnetite layers after immersion treatment and the flakes that were taken from an SG tube. Numerous small pores with diameters between 1 and 2 μm were observed on the cross section of the magnetite layers (Figure 6a). In addition, numerous pores were present on both the tube and water surfaces of the flakes. The numerous pores with diameters between 0.1 and 3 μm on the tube surface are associated with bubbles that occurred on the tube surface. However, on the water side of the flakes, large pores with diameters between 5 and 7 μm were observed, and they are believed to be associated with the steam chimneys. Large pores over the tube surfaces are thought to provide the main route for escaping the steam produced by boiling close to the tube wall [28].

**Figure 7:**
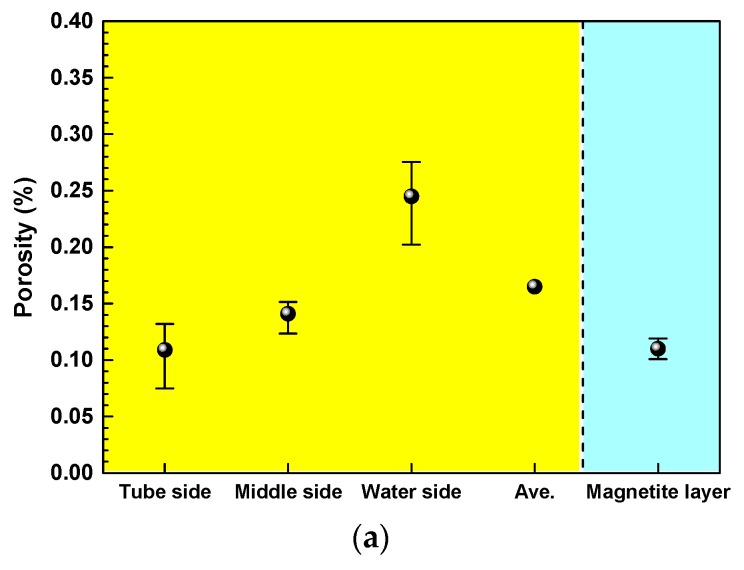

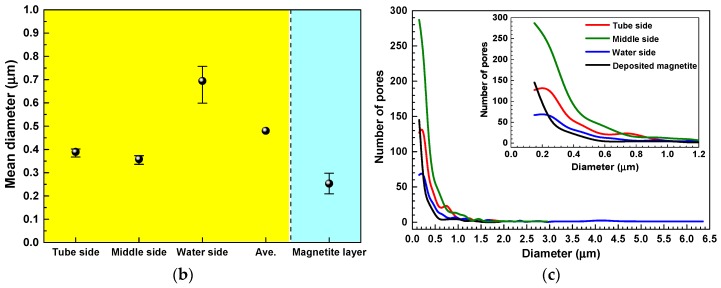
Porosity, pore size, and pore distribution of the magnetite layer after immersion in an alkaline solution for 50 days at 25 °C and flakes collected from SG tubes: (**a**) porosity, (**b**) mean pore size, and (**c**) pore distribution.

Figure 7 presents the porosity, pore size, and pore size distribution of the electrodeposited magnetite layer and flake samples. As shown in Figure 7a, the porosity of the flake samples increased from tube side to water side. This result may be associated with the bubble growth and steam chimneys from the tube side to the water side. Although the porosity of the electrodeposited magnetite layer was quite different from that of the water side of the flake samples, the porosity of the electrodeposited magnetite was almost the same as that of the tube side and middle of the flake samples, with about 11~13% porosity. In addition, the porosity of the electrodeposited magnetite layer was not much different from the average porosity of whole (tube, middle, water sides) flake sample. The pore mean diameter of the water side of the flake samples was about 1.7 times larger than that of tube and middle sides of the flake samples (Figure 7b). Although the mean pore diameter of the water side of the flakes was much larger than that of the electrodeposited magnetite layer, the mean pore diameter of the tube and middle sides of the flake samples was not much different from that of the electrodeposited magnetite layer. As seen in Figure 7c, the water side of the flake samples showed an increased number of coarse pores, and the maximum pore diameter was about 6.35 μm. In addition, the maximum pore diameters of the tube and middle sides of the flakes and the electrodeposited magnetite were almost the same (about 2.05 μm).

### 3.3. Consideration of Applications in Nuclear Research Using Porous Magnetite Layers

In this work, to overcome the limitations of dense magnetite layers in previous research, porous magnetite layers were newly produced on Alloy 690TT substrates by the electrodeposition method and immersion in an alkaline solution using a magnetite reductive reaction. In the secondary water systems of PWRs, the content of dissolved oxygen is maintained below 10 ppb and magnetite is formed under the condition of 10 ppb oxygen content. However, in this electrodeposition system, magnetite was produced by cathodic deposition by electrochemical reduction of Fe(III) complexed with TEA. Although Fe(III) complexed with TEA was not sensitive to oxygen from the air, the oxygen content of the electrodeposition system was quite different from that of the secondary water system. The formation processes of porous magnetite through electrodeposition and in the secondary water systems of actual PWRs are different. However, based on the results obtained in this work, the phase fraction, morphology, porosity, and chemical composition of the electrodeposited magnetite sample were quite similar to those of the flake samples that were collected from a PWR. Therefore, this newly modified method is appropriate to simulate the real corrosion products that are formed on SG tubes in nuclear power plants.

In addition, the remaining problem in this method is that it needs a relatively long process time to produce the porous magnetite layer. The process time can be effectively reduced using wet-chemical etching methods. Using EDTA-based solutions will be a potential way. This solution has been widely used for chemical cleaning to remove the magnetite sludge in SG of PWRs [29,30,31,32]. Thus, it is expected that the method using EDTA-based solutions can rapidly produce the more porous magnetite layers owing to the increased dissolution rate of dense magnetite layers. Another way is to control the solubility of magnetite. The solubility of magnetite shows a maximum peak at a temperatureof around 150 °C, and decreases as the temperature deviates from the peak temperature [33]. That is, the dissolution rate of magnetite is highest at about 150 °C, resulting in the increased formation of pores inside the magnetite layer. Furthermore, the solubility of magnetite is increased with a decreasing the pH value [33]. Therefore, it is also expected that controlling these two factors can reduce the process time to make the porous magnetite layer.

In SG systems, porous magnetite is the main corrosion product that is formed on the SG tubing and other support structures. The heat transfer efficiency of SG tubes from the primary side to the secondary side decreases due to the accumulation of porous magnetite. In addition, porous magnetite deposits accelerate the corrosion of SG tubes. In general, an increase in temperature is expected within the magnetite tha is deposited on the outer surface of SG tubes due to the deterioration of heat transfer efficiency. Increasing the temperature on the outer surface of the SG tubes leads to an increase in the solubility of impurities, forming a corrosive environment within the porous magnetite deposits. In particular, aggressive ionic species, such as chloride and sulfate ions, have been reported to accelerate the corrosion degradation of nickel-based SG tubes [34]. Therefore, the heat transfer reduction and corrosion behavior of SG tubes covering porous magnetite deposits is a highly concerning issue.

**Figure 8:**
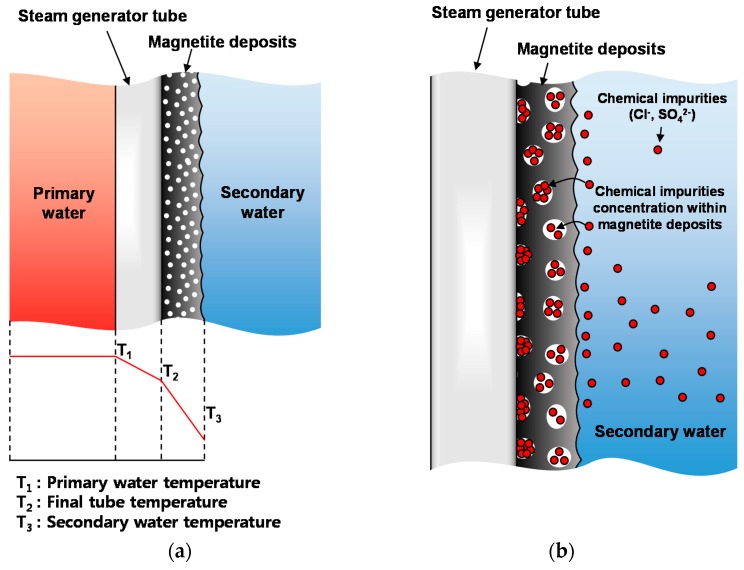
Schematics of various applications in nuclear research using porous magnetite layer electrodeposited on SG tubes: (**a**) heat transfer deterioration and (**b**) corrosion acceleration due to the concentration of chemical impurities.

However, there are difficulties that are associated with dense magnetite layers regarding the investigation of the above-mentioned problems. The heat transfer efficiency of dense magnetite layers differs from that of porous magnetite deposits. Also, aggressive ionic species, such as chloride and sulfate ions, cannot be accumulated in dense magnetite layers. However, as shown in Figure 8a, porous magnetite layers that are produced by the new method can be used for studying the variation of heat transfer according to the thickness of magnetite accumulated on SG tubes. Furthermore, it can be used to investigate the corrosion rate and the behavior of SG tubes in experimental solutions containing aggressive ionic species (Figure 8b). In our research group, corrosion experiments are ongoing to validate the effect of impurities on the corrosion rate of porous magnetite deposited on SG tubing in secondary water systems at temperatures of up to 280 °C.

This method is a new approach to simulating the porous magnetite deposited on SG tubes in nuclear power plants. This method is also a very simple and easy way to simulate porous magnetite at temperatures below 80 °C. In consideration of these advantages, porous magnetite specimens that are produced by the proposed method could be usefully applied in various nuclear research fields.

## 4. Conclusions

The objective of this study was to simulate porous magnetite that is deposited on Alloy 690TT SG tubes. Magnetite layers were electrodeposited on Alloy 690TT substrates in an Fe(III)-TEA solution. After electrodeposition, the magnetite layers were immersed to simulate the porous magnetite deposits in an alkaline solution for 50 days at room temperature. As a result, porous magnetite layers were produced by the reductive reaction. The porous magnetite layer electrodeposited at −1.05 V_SCE_ for 10 h is appropriate to simulate real corrosion products because the structure, morphology, thickness, and porosity of magnetite are similar to those of SG flakes. This method is a simple way to simulate the porous magnetite deposited on the tubing of secondary systems. Using porous magnetite layers, various aspects of nuclear research, such as heat transfer characteristics and the corrosion acceleration of porous magnetite that is deposited on SG tubes, can be investigated.

## Figures and Tables

**Table 1:** Chemical composition of Alloy 690TT (wt %).

Ni	Cr	Fe	C	Si	Mn	Ti	Al
Bal.	28.0	10.2	0.02	0.1	0.3	0.1	0.1

**Table 2:** Elemental analysis results of flake samples taken from an steam generator (SG) tube of an actual nuclear power plant obtained by X-ray fluorescence (XRF) spectrometry (wt %).

Elements	Sample 1	Sample 2
Fe	92.7	93.9
Mn	2.32	2.77
Ni	2.92	2.05
Cr	0.68	0.41
Cu	0.45	0.43
Zn	0.59	0.37
Ti	0.23	0.40
Al	0.10	0.28

**Table 3:** Lattice constant of electrodeposited magnetite and flake samples calculated by JADE9 software and PDF data.

Phase	Space Group	Lattice Constant (Å)	PDF Number
Magnetite Layers	Fd-3m (227)	8.402–8.419	01-080-6406
Flakes	Fd-3m (227)	8.407–8.411	01-086-1360
Magnetite (Fe_3_O_4_)	Fd-3m (227)	8.394	01-076-4112
		8.399	01-079-0418
		8.400	01-076-1849
		8.405	01-080-7683
		8.419	01-080-6405
		8.432	01-080-6406
Trevorite (NiFe_2_O_4_)	Fd-3m (227)	8.308	01-082-8442
		8.337	01-089-4927
		8.338	01-074-2081
		8.339	01-078-6781
		8.351	01-081-8428
		8.357	01-071-3850
Chromite (FeCr_2_O_4_)	Fd-3m (227)	8.377	01-075-3312
		8.378	01-089-2618
		8.383	01-080-6393
		8.390	01-089-3855

**Table 4:** Full-width at half-maximum (FWHM) and crystallite size of electrodeposited magnetite and flake samples calculated by JADE9 software and Sherrer’s formula.

Peaks	Magnetite Layer		Flake Samples	
	FWHM (°)	Crystallite Size (nm)	FWHM (°)	Crystallite Size (nm)
D_220_	0.097	84.7	0.147	55.9
D_311_	0.118	70.8	0.158	52.7
D_400_	0.109	78.0	0.172	49.8
D_422_	0.101	88.3	0.191	46.5
D_511_	0.123	73.2	0.210	43.0
D_440_	0.111	84.0	0.227	40.9
Average	0.110	79.8	0.184	48.1

**Table 5:** Chemical composition of the electrodeposited magnetite and flake samples analyzed by SEM-EDS (at. %).

Samples	Point	Chemical Composition				Structure
		Fe	Ni	Cu	O	
Electrodeposited Magnetite	1	42.5	-	-	57.5	Magnetite
	2	43.1	-	-	56.9	
Flakes	3	40.3	-	-	59.7	Magnetite
	4	37.1	17.9	-	45.0	Trevorite
	5	5.7	2.1	86.4	5.8	Metallic Cu

## References

[1] Ramesh C., Murugesan N., Prince A.A.M., Velmurugan S., Narasimhan S.V., Ganesan V. (2001). Applied of polymer electrolyte based hydrogen sensor to study corrosion of carbon steel in acid medium. Corros. Sci..

[2] Prince A.A.M., Velmurugan S., Narasimhan S.V., Ramesh C., Murugesan N., Raghavan P.S., Gopalan R.J. (2001). Dissolution behaviour of magnetite film formed over carbon steel in dilute organic acid media. J. Nucl. Mater..

[3] Paine J.P.N., Hobart S.A., Sawochka S.G. Predicting steam generator crevice chemistry. Proceedings of the 5th International Symposium on Environmental Degradation of Materials in Nuclear Power Systems-Water Reactors.

[4] Millet P.J., Fenton J.M. A detailed model of localized concentration processes in porous deposits of SGs. Proceedings of the 5th International Symposium on Environmental Degradation of Materials in Nuclear Power Systems-Water Reactors.

[5] Sakai T., Senjuh T., Aoki K., Shigemitsu T., Kishi Y. Lead induced stress corrosion cracking of Alloy 600 and 690 in high temperature water. Proceedings of the 5th International Symposium on Environmental Degradation of Materials in Nuclear Power Systems-Water Reactors.

[6] Yonezawa T., Onimura K., Sasaguri N., Kusakabe T., Nagano H., Yamanaka K., Minami T., Inoue M. Effect of heat treatment on corrosion resistance of Alloy 690. Proceedings of the 2nd International Symposium on Environmental Degradation of Materials in Nuclear Power Systems-Water Reactors.

[7] Paine J.P.N., Pathania R.S., Shoemaker C.E. Effect of caustic environment on intergranular attack and stress corrosion cracking of Alloy 600. Proceedings of the 3th International Symposium on Environmental Degradation of Materials in Nuclear Power Systems-Water Reactors.

[8] Gonzalez F., Spekkens P. Corrosion of Inconel 600 under steam generator sludge piles. Proceedings of the 4th International Symposium on Environmental Degradation of Materials in Nuclear Power Systems-Water Reactors.

[9] Saint P., Slama G. Steam generator materials degradation. Proceedings of the 5th International Symposium on Environmental Degradation of Materials in Nuclear Power Systems-Water Reactors.

[10] Kawamura H., Hirano H. Intergranular attack and stress corrosion cracking propagation behavior of Inconel 600 in high temperature water. Proceedings of the 6th International Symposium on Environmental Degradation of Materials in Nuclear Power Systems-Water Reactors.

[11] Sapieszko R.S., Matijevic E. (1980). Preparation of well-defined colloidal particles by thermal decomposition of metal chelates. I. Iron oxides. J. Colloid Interface Sci..

[12] Kothari H.M., Kulp E.A., Limmer S.J., Poizot P., Bohannan E.W., Switzer J.A. (2006). Electrochemical deposition and characterization of Fe_3_O_4_ films produced by the reduction of Fe(III)-triethanolamine. J. Mater. Res..

[13] Kulp E.A., Kothari H.M., Limmer S.J., Yang J., Gudavarthy R.V., Bohannan E.W., Switzer J.A. (2009). Electrodeposition of epitaxial magnetite films and ferrihydrite nanoribbons on single-crystal gold. Chem. Mater..

[14] Gudavarthy R.V., Gorantla S., Mu G., Kulp E.A., Gemming T., Eckert J., Switzer J.A. (2011). Epitaxial electrodeposition of Fe_3_O_4_ on single-crystal Ni(111). Chem. Mater..

[15] Goujon C., Pauporté T., Mansour C., Delaunary S., Bretelle J.L. Fouling of steam generator tubes in nuclear power plants: Laboratory tests to reproduce oxides deposition. Proceedings of the International Conference on Heat Exchanger Fouling and Cleaning.

[16] Goujon C., Pauporté T., Mansour C., Delaunary S., Bretelle J.L. (2015). Electrochemical deposition of thick iron oxide films on nickel based superalloy substrates. Electrochim. Acta.

[17] Duan H., Chen X., Li B., Liang J. (2010). Growth morphology study of cathodically electrodeposited Fe_3_O_4_ thin films at elevated temperatures. Mater. Res. Bull..

[18] Manahan M.P. (1990). Mechanical behaviour of magnetite from the Oconee-2 and TMI-1 steam generators using miniaturized specimen technology. J. Mater. Sci..

[19] Cullity B.D. (1978). Elements of X-ray Diffraction.

[20] Jeon S.H., Song G.D., Hur D.H. (2016). Effects of deposition potentials on the morphology and structure of iron-based films on carbon steel substrate in an alkaline solution. Adv. Mater. Sci. Eng..

[21] Laronge T.M., Ward M.A. (1999). The Basics and Not So Basics of Water Corrosion Processed Altered by Flow Changes. Proceedings of the Corrosion 1999 conference.

[22] Bobinson J.O., Drews T. (1999). Resolving Flow-Accelerated Corrosion Problems in the Industrial Steam Plant. Proceedings of the Corrosion 1999 Conference.

[23] Vepsäläinen M., Saario T. (2010). Magnetite Dissolution and Deposition in NPP Secondary Circuit.

[24] Lister D.H., Lang L. A Mechanistic Model for Predicting Flow-Assisted and General Corrosion of Carbon Steel in Reactor Primary Coolant. Proceedings of the CHIMIE 2002 International Conference Water Chemistry in Nuclear Reactor Systems, Operation Optimum and New Developments.

[25] Jung K.S., Sung K.W. (2010). Magnetite: Structure, Properties and Applications.

[26] Lemos V.P., Costa M.L.D., Lemos R.L., Faria M.S.G.D. (2007). Vivianite and siderite in lateritic iron crust: An example of bioreduction. Quim. Nova.

[27] Al-Mayouf A.M. (2002). Electrochemical Investigation of Magnetite Reductive Dissolution in Aqueous Solutions. Corrosion.

[28] Mclean V. (1996). Characterization of PWR Steam Generator Deposits.

[29] Schneidmiller D., Stiteler D. (1983). Steam Generator Chemical Cleaning Process Development.

[30] Jevec J.M., Leedy W.S. (1983). Chemical Cleaning Solvents and Process Testing.

[31] Helyer M.H., Glaves C.L. (1986). Chemical Cleaning of PWR Steam Generator Sludge Piles.

[32] Hur D.H., Chung H.S., Kim U.C. (1995). Corrosion behaviors during the iron removal process for chemical cleaning of nuclear steam generators. J. Nucl. Mater..

[33] Dooley R.B. (2008). Flow-accelerated corrosion in fossil and combined cycle/HRSG plants. Power Plant Chem..

[34] Steahle R.W., Gorman J.A. (2003). Quantitative assessment of submodes of stress corrosion cracking on the secondary side of steam generator tubing in pressurized water reactors: Part. 1. Corrosion.

